# Clinical features and outcome in patients with osseomuscular type of Wilson’s disease

**DOI:** 10.1186/s12883-017-0818-1

**Published:** 2017-02-17

**Authors:** Hao Yu, Juan-Juan Xie, Yu-Chao Chen, Qin-Yun Dong, Yi Dong, Wang Ni, Zhi-Ying Wu

**Affiliations:** 1 Department of Neurology and Research Center of Neurology in Second Affiliated Hospital, and the Collaborative Innovation Center for Brain Science, Zhejiang University School of Medicine, 88 Jiefang Rd, Hangzhou, 310009 China; 2 Department of Neurology and Institute of Neurology, First Affiliated Hospital, Fujian Medical University, Fuzhou, China; 3 Department of Neurology and Institute of Neurology, Huashan Hospital, Shanghai Medical College, Fudan University, Shanghai, China; 4 Joint Institute for Genetics and Genome Medicine between Zhejiang University and University of Toronto, Zhejiang University, Hangzhou, China

**Keywords:** Arthralgia, ATP7B, Copper, Deformity, Osteoarthritis

## Abstract

**Background:**

Wilson﻿'s disease ﻿with﻿ osseomuscular type is a rare condition, which often lacks typical hepatic and neurological symptoms and causes misdiagnoses easily. During the past 10 years, eight Chinese patients of osseomuscular type of Wilson's disease were identified in our clinic.

**Methods:**

Clinical information was gathered from medical records and follow-ups. The genetic testing was performed in each patient. Serum ceruloplasmin, Kayser-Fleischer rings, liver function, brain magnetic resonance imaging and abdominal ultrasonography were also evaluated.

**Results:**

The median age of onset is 12 years of age. The patients had their initial musculoskeletal conditions with arthralgia or joint deformity, while the hepatic or neurologic signs were minimal. Most patients (6/8) eventually developed clinical neurological symptoms afterwards with a median interval of 36 months. All of them had normal liver function and low serum ceruloplasmin (<0.1 g/L). Most patients (6/8) present with Kayser-Fleischer rings and abnormal hepatic ultrasonography. The arthralgia was resolved with copper chelation therapy.

**Conclusions:**

Wilson’s disease with osseomuscular type occurs without typical hepatic or neurological symptoms, which makes the clinical diagnosis challenging. Serum ceruloplasmin, abdominal ultrasonography, ophthalmic examination and genetic testing help to establish the diagnosis. Early diagnosis can initiate an effective treatment and prevent the further damage.

## Background

Wilson’s disease (WD), also known as hepatolenticular degeneration, is an autosomal recessive disease (incidence 1/30,000) characterized by Kayser-Fleischer rings of cornea (K-F rings) and multisystem damage, including liver cirrhosis, neurological symptoms and musculoskeletal deformity. The disease is caused by mutations in the *ATP7B* gene, which encodes a copper-transporting ATPase in the liver. The deficiency in the enzyme activity can lead to a toxic copper accumulation in multiple organs, which may be responsible for its wide variety of symptoms [1].

Despite wide varieties of clinical manifestations, WD patients typically present with liver dysfunction or neurological disorders. Musculoskeletal abnormalities, including premature osteoarthritis, skeletal deformity and pathological bone fractures, can be occasionally found in WD patients with hepatic or neurologic type [2, 3], but very rare as initial symptoms (incidence ~2% in Chinese population) [4]. These conditions, also known as “osseomuscular type” of WD, often lack typical hepatic and neurological symptoms and cause misdiagnoses easily [5, 6].

During the past 10 years, we have collected and identified eight Chinese WD patients with osseomuscular type in our clinic. Their genotypes, clinical manifestations, biochemical parameters and outcome of treatment are reported herein, aiming to improve early diagnosis and therapy with this condition.

## Methods

### Subjects

All eight WD patients were diagnosed by genetic testing. The degree of osseomuscular symptoms was based on the published assessment scale (0: Normal. 1: Abnormal skeletal X-ray; asymptomatic.2: Difficulty in ADL but independent. 3: Requires help in ADL. 4: Dependent on others for ADL. 5: Fracture or bedbound) [7]. In addition, ceruloplasmin, K-F rings, liver function, brain magnetic resonance imaging (MRI) and abdominal ultrasonography were also evaluated.

### Genotype analysis

Genomic DNA was extracted from peripheral EDTA-treated blood by Blood Genomic Extraction Kit (Qiagen, Hilden, Germany). The Sanger sequencing of *ATP7B* was performed with a procedure described previously [8].

## Results

### Mutation analysis

All eight patients carried biallelic mutations, and seven of them were compound heterogeneous (Table 1). The most frequent mutation was p.R778L (6/16), which was also the most common mutation of *ATB7B* in Chinese population [9].

**Table 1:** Genotype and clinical course in eight cases of WD with osseomuscular type

No	Gender	*ATP7B* mutations	Onset Age (years)	Osseomuscular abnormalities (Degree^a^)	Neurological symptoms	Interval from O to N (months)	Orthopedic Surgery	Interval from S to N (months)
1	F	p.A874V + p.P992L	10	Genu valgum (3)	Dysarthria, difficult with writing, musk face	8	Yes	2
2	M	p.M769Hfs*26 + p.R919G	17	Arthralgia in knee joints (2)	Dysarthria, tremor	60	No	-
3	M	p.R778Q + p.I930del	12	Arthralgia in knee joints (2)	Normal	-	No	-
4	F	p.E332* + p.R778L	10	Genu varum (3)	Dysarthria, bradykinesia	18	Yes	15
5	F	p.R778L + p.I1148T	13	Genu varum, femoral head necrosis, dislocation of the left shoulder (3)	Tremor	36	Yes	36
6	F	p.R778L + p.R778L	15	Talipes equinovarus (3)	Tremor writer’s cramp	N/A	Yes	<2
7	M	p.R778L + p.G943D	12	Arthralgia in knee joints (4)	Tremor, dysarthria	84	No	-
8	M	p.R778L + p.V1106I	3	Arthralgia in hip and knee joints, dysplasia of thoracic vertebrae (4)	Normal	N/A	No	-

﻿*Translation te﻿rmination c﻿o﻿don, ﻿*O* osseomuscular symptoms, *N* neurological symptom, *S* surgery, *N/A* not available

^a^Based on the published assessment scale for osseomuscular symptoms [7]

### Clinical manifestation

All of patients denied WD family histories. The median age of onset is 12 years of age (range 3–17 years). Their complains were all about musculoskeletal problems such as arthralgia and joint deformity (Table 1). The abnormalities include arthralgia of the knees in four patients, genu varum in two patients, arthralgia of hip joints in one patient, genu valgum in one patient and talipse equinovarus in one patient. The median assessment score of osseomuscular symptoms is 3 (range 2–4).

As an example, Patient 8 is a 13-year-old boy with a 10-year history of walking difficulty. After a hernia surgery at 3 years of age, he felt knees and ankles sore after taking a long walk. The condition progressed slowly and he got a hip pain at 12 years of age. The pain was aggravated by walking and rapidly made him unable to walk any more. Physical examination revealed a severely limited movement of bilateral hip joint in lower limbs. The motion range of his hip joint was only about 25° (flexion 45° to 70°). Excessive extension or flexion would elicit intense and sharp pain immediately, so the patient could never straighten his legs. When standing and walking, he had to keep a “crouching” posture with bent hips, and steady himself with hands on knees. His condition had been considered as a rheumatic disease for many years and diagnosed as “arthralgia” or “osteoarthritis” in many hospitals. However, the diagnosis was indefinite and he only received some anti-inflammatory therapy.

Similar to Patient 8, all the other patients also sought for medical attention in orthopedics or rheumatology firstly, due to absence of neurological and hepatic symptoms. Moreover, four patients (Patient 1, 4, 5, 6) with deformities subsequently underwent the orthopedic surgeries. However, the orthopedic surgery did not correct the skeletal abnormalities and neurological deterioration ensued. Patients 1 and 6 showed neurological symptoms shortly after surgeries (<2 months) and the other two developed neurological symptoms after 15 and 36 months respectively (Table 1).

Despite the absence of neurological complaints initially, it is noteworthy that most patients (6/8) eventually developed neurological symptoms afterwards with a median interval of 36 months (range 8–84 months), including tremor, dysarthria, writing difficulty and bradykinesia (Table 1). Then it is the moment when most diagnoses of WD were taken into consideration in these patients. The interval between the onset of osseomuscular symptoms and establishment of the diagnosis was at least 10 months (Patient 1) and could reach as long as 10 years (Patient 8). The neurological symptoms included tremor in four patients, dysarthria in four patients, writing difficulty in two patients and bradykinesia in one patient. Patient 1 also manifested psychiatric symptoms.

### Accessory examination

The K-F ring, known as a specific sign in WD [1], was present in most patients (6/8) (Table 2). However, since most patients had developed the neurological symptoms when examining, the initial rate of K-F rings might be lower.

**Table 2:** Clinical findings and treatment outcomes in eight cases of WD with osseomuscular type

No	K-F ring	Neurologic Examination			CP (g/L)	Involvement on brain MRI	Abdominal ultrasonography	Outcomes of Chelation Therapy (Degree^a^)
		Strength	Tone	Reflex				
1	+	UE & LE 5	UE ↑	(++)	<0.02	Basal ganglia	Hepatic diffuse disease	(9 months after treatment) Dystonia in the right hand partially relieved; blurred speech persist; walking better (2)
2	+	UE & LE 5	Normal	(++)	0.02	Basal ganglia and brain stem	Hepatic diffuse disease, splenomegaly	(3 months after the treatment) Pain relieved with improved speech (0)
3	+	UE & LE 5	Normal	(++)	<0.02	Normal	Hepatic diffuse disease, splenomegaly	(14 months after treatment) Pain relieved (0)
4	+	UE & LE 5	UE & LE ↑	(++)	0.07	N/A	Hepatic diffuse disease	(after 23 months of inconsecutive treatment) No obvious improvement (3)
5	N/A	UE & LE 5	UE ↑	UE & LE (+++)	0.03	Basal ganglia and brain stem	Hepatic diffuse disease, splenomegaly	N/A
6	+	UE & LE 5	UE & LE ↓	(++)	0.03	N/A	Normal	N/A
7	+	UE 5; pLE 4; dLE 4-	UE & LE ↓	Knee (+++)	0.02	N/A	Hepatic diffuse disease, splenomegaly	N/A
8	-	UE 4; LE 4-	LE ↑	Knee (+++)	0.05	Normal	Increased echogenicity	(6 months after treatment) Pain relieved with improved joint motion (2)

*UE* upper extremity, *LE* lower extremity, *dLE* distal lower extremity, *pLE* proximal lower extremity, *CP* ceruloplasmin, *N/A* not available

^a^Based on the published assessment scale for osseomuscular symptoms [7]

Serum ceruloplasmin concentration was less than 0.1 g/L (reference: 0.2–0.4 g/L) in all patients. No significant abnormalities in liver function were observed, including aminotransferase, bilirubin and albumin parameters. However, the ultrasonography showed features of chronic hepatic diseases in most patients (6/8) (Table 2). All the patients were evaluated as Class A based on Child-Pugh score.

Among the five patients taking the brain MRI, four showed different degree of abnormal MRI signals. Abnormal T2 signals were discovered in three cases in basal ganglia, and two cases in the brain stem (Table 2).

**Fig. 1:**
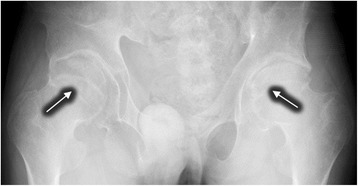
The plain film showed hip osteoarthritis with narrowing joint space, incomplete articular surface and swelling joint capsule (*Arrows*)

**Fig. 2:**
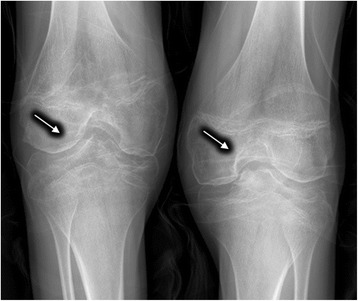
The knee plain film of knee joints showed degenerative changes with articular surface hyperosteogeny and swelling joint capsule (*Arrows*)

**Fig. 3:**
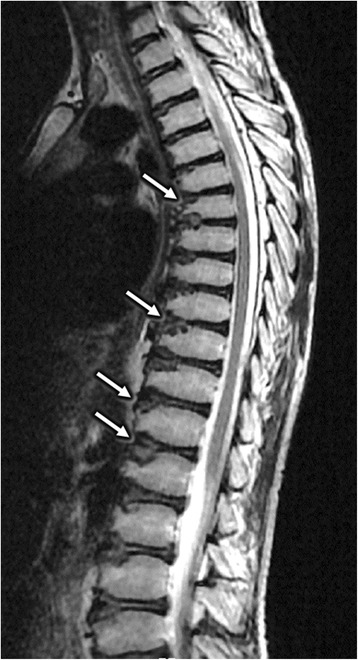
The thoracic MRI showed the dysplasia of thoracic vertebrae, which had bullet-like shapes with focal cartilage defects at the front edge (*Arrows*)

For Patient 8, despite a decreased serum ceruloplasmin (0.05 g/L), he had no K-F rings, no abnormalities in brain MRI or hepatic ultrasonography. His X-rays films showed bilateral hip osteoarthritis (Fig. 1) and knee degenerative changes (Fig. 2). Notably, the thoracic MRI incidentally found the dysplasia of thoracic vertebrae (Fig. 3). Due to the absence of hepatic, neurologic symptoms and K-F ring, his diagnosis of WD was confirmed by genetic testing (Table 1).

### Outcome of treatment

After the establishment of diagnosis, all patients received a copper chelator treatment with D-penicillamine, dimercaptosuccinic acid (DMSA) or dimercaptopropane sulfonate (DMPS). No serious related adverse reaction was observed in these patients. The patients responded generally well to the therapy. The arthralgia was all resolved and neurological symptoms showed different degrees of improvement (Table 2). However, the improvements in bone deformity were minimal.

The Patient 8 improved remarkably by treatment. After one course of intravenous DMPS (0.25 g q.d. for 6 days) and 3 months of oral DMSA (0.5 g b.i.d.), the patient felt less painful to move his legs and easier to stand up from a chair. The physical examination confirmed an improved movement of his hip joint. The range of motion was able to reach a degree of 35°, which increased by 40% compared with the condition 3 months ago. After 6 month of treatment, the range increased to a degree of 80° and the patient can stand and walk without the assistance of his hand.

## Discussion

WD is one of the limited genetic diseases that can be clinically cured by timely treatment. The osseomuscular type of WD begins without typical hepatic and neurological symptoms, which makes the diagnosis challenging. Most patients are never considered WD until the neurological symptoms emerged. However, some typical signs were present in our patients, such as K-F rings or abnormal liver ultrasonography. Moreover, serum ceruloplasmin is a sensitive test in WD patients even without any hepatic and neurological signs. WD should be highly suspected when ceruloplasmin is below 0.1 g/L, but lower levels can also occur with aceruloplasminemia, renal or enteric protein loss, liver disease and heterozygotes for WD [10]. Therefore, if the patient has a low serum ceruloplasmin without K-F rings, the diagnosis should be based on combination of other tests. Genetic testing is confirmatory and non-invasive, and also convenient for screening family members.

The pathogenic mechanisms underlying the musculoskeletal damage are not clear. However, it may be a result of abnormal bone metabolism. Abnormal copper deposit are found in multiple organs and structures of WD patients, including the bone, in which the copper concentration increases about four times [11]. The radiological changes of arthropathy have been frequently encountered among WD patients [11–13]. Moreover, the pathological findings suggested a chronic inflammatory process or degradation of collagen and protein secondary to a deposition of copper [14]. Besides, the calcium regulation disorder also makes the bone more vulnerable to other stimuli, which can result from vitamin D insufficiency. The hepatic damage in WD can impede the conversion of vitamin D to its active form, thus impaired absorption of calcium and led to osteoporosis and bone deformity [15]. The similar effects caused by the damage of kidney and parathyroid gland in WD patients are also reported [16, 17]. These pathological conditions may be corrected by timely chelation therapy and hormone supplement.

Dystonia, which is characterized by “sustained or intermittent muscle contractions causing abnormal, often repetitive, movements, postures or both”, can develop into contracture and joint deformity in severe cases [18]. These conditions usually demonstrate spasticity with markedly increased muscle tone. In our patients, half of them (Patient 1, 4, 5, 8) had focal or general increase tones. However, they took 8–36 months to develop the other neurological symptoms, such as dysarthria, which usually arose with dystonia in other WD patients. In addition, Patient 8 never developed any other neurological signs in his 10-year course, which made it. Therefore, it remains obscure whether the osseomuscular type of WD is a result of long-term dystonia or a natural course of osteopathy.

## Conclusions

Although most WD patients with osseomuscular type will develop neurological symptoms, it is easily misdiagnosed when it only manifests as joint pain or bone deformity. A long-term misdiagnosis may lead to irreversible damage to the musculoskeletal system, such as a permanent deformity, which often requires surgical intervention and has a poor response to the drug. Therefore, if a child or adolescent presents with unexplained musculoskeletal problems, the possibility of WD should be considered. Serum ceruloplasmin, abdominal ultrasonography, ophthalmic examinations and genetic testing will help to establish the diagnosis. Early diagnosis can initiate an effective treatment and prevent the further damage.

## Abbreviations

DMPS: Dimercaptopropane sulfonate
DMSA: Dimercaptosuccinic acid
K-F ring: Kayser-Fleischer ring
MRI: Magnetic resonance imaging
WD: Wilson’s disease
